# Supplementary material for: 3D Cell Culture Models Demonstrate a Role for FGF and WNT Signaling in Regulation of Lung Epithelial Cell Fate and Morphogenesis
Source: Front Cell Dev Biol. 2020 Jul 21;8:574. doi: 10.3389/fcell.2020.00574 (PMC7396690; doi:10.3389/fcell.2020.00574)
Supplement: Supplementary file 1 [file Data_Sheet_1.pdf]

Supplementary Material

Supplementary Methods

Transmission electron microscopy

The lungospheres that formed in non-adherent conditions or in 3D Matrigel were washed in 0.1 M cacodylate buffer [0.1 M sodium cacodylate in distilled water (Sigma/Merck)], fixed with 3% glutaraldehyde for 1 h and postfixed in 1% OsO_4 for 50 min. The cells were washed in cacodylate buffer, embedded in 1% agar blocks, dehydrated in increasing series of ethanol (50, 70, 96, and 100%), treated with 100% acetone, and embedded in Durcupan resin (Sigma/Merck). Ultrathin sections were prepared using LKB 8802A Ultramicrotome, stained with uranyl acetate and Reynold's lead citrate (Sigma/Merck), and examined with FEI Morgagni 286(D) transmission electron microscope.

Supplementary Figures and Tables

Supplementary Figures

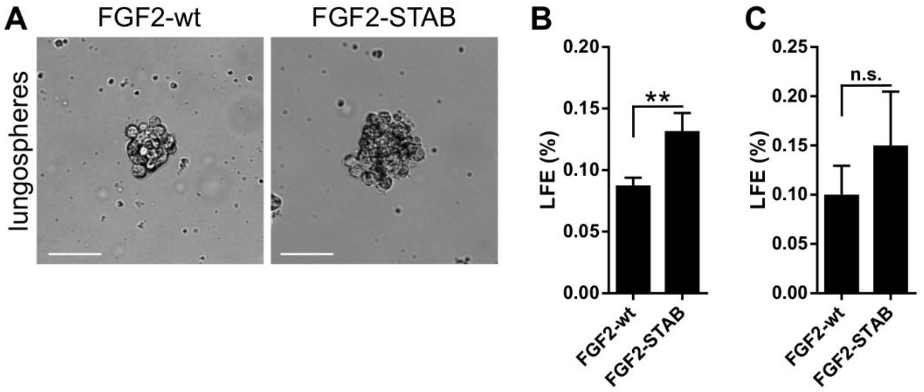

Supplementary Figure 1. Hyperstable FGF2 promotes formation of lungospheres with higher efficiency than wild-type FGF2. (**A**) Representative photographs of lungospheres formed in the presence of FGF2-wt and FGF2-STAB. Scale bars, 100 µm. (**B, C**) The efficiency of primary (**B**) and secondary (**C**) lungosphere formation (LFE). The plots show mean + SD; n = 4-6. **P<0.01 (Student's t-test).

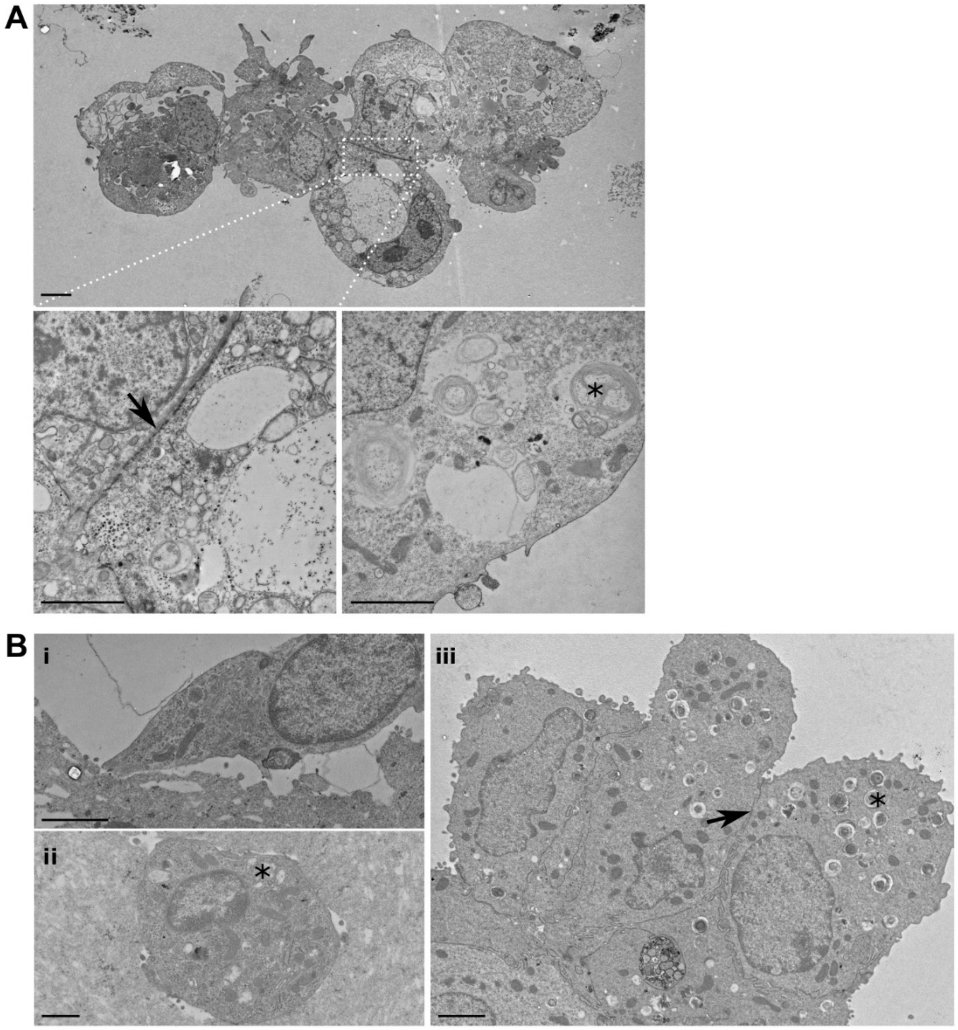

Supplementary Figure 2. Ultrastructural analysis of lungospheres and lung organoids. (**A**) Transmission electron microscopy (TEM) photograph of a lungosphere formed from unsorted lung epithelial cells in non-adherent conditions. The cells attach to each other by intercellular junctions (indicated by an arrow). Some cells contain lamellar bodies (indicated by a star). (**B**) TEM photographs of cells from lung organoids cultured in 3D Matrigel. The photographs show (i) ATI-like cell, (ii) ATII-like cell, and (iii) club cell-like cell. Arrow, intercellular junctions; stars, lamellar bodies. Scale bars, 2 μm.

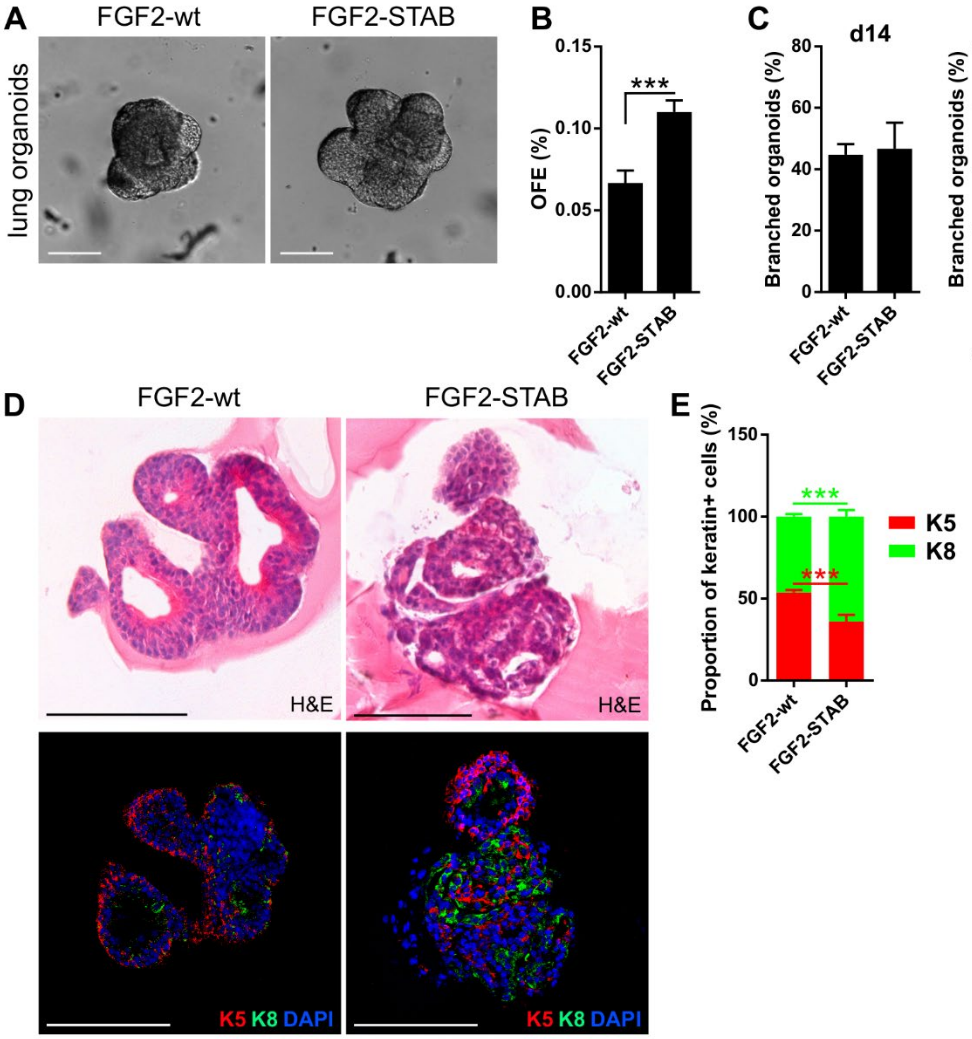

Supplementary Figure 3. Hyperstable FGF2 promotes formation of lung organoids with higher efficiency than wild-type FGF2. (**A**) Representative photographs of lung organoids formed in the presence of FGF2-wt and FGF2-STAB. Scale bars, 100 µm. (**B**) The efficiency of primary lung organoid formation (OFE), shown as mean + SD; n = 3. ***P<0.001 (Student's t-test). (**C**) Branching efficiency (%) of lung organoids on day 14 and 21 of culture, shown as mean + SD; n = 3. (**D**) Hematoxylin-eosin (H&E) and immunofluorescence staining of lung organoid sections for keratin 5 (K5) and keratin 8 (K8). Scale bars, 100 µm. (**E**) The plot shows proportion of K5 positive (+) or K8+ cells in the total number of keratin+ (sum of K5+ and K8+ cells) cells in the organoid sections as mean + SD; n = 3, N = 2-3 organoids per experiment. ***P<0.001 (one-way ANOVA).

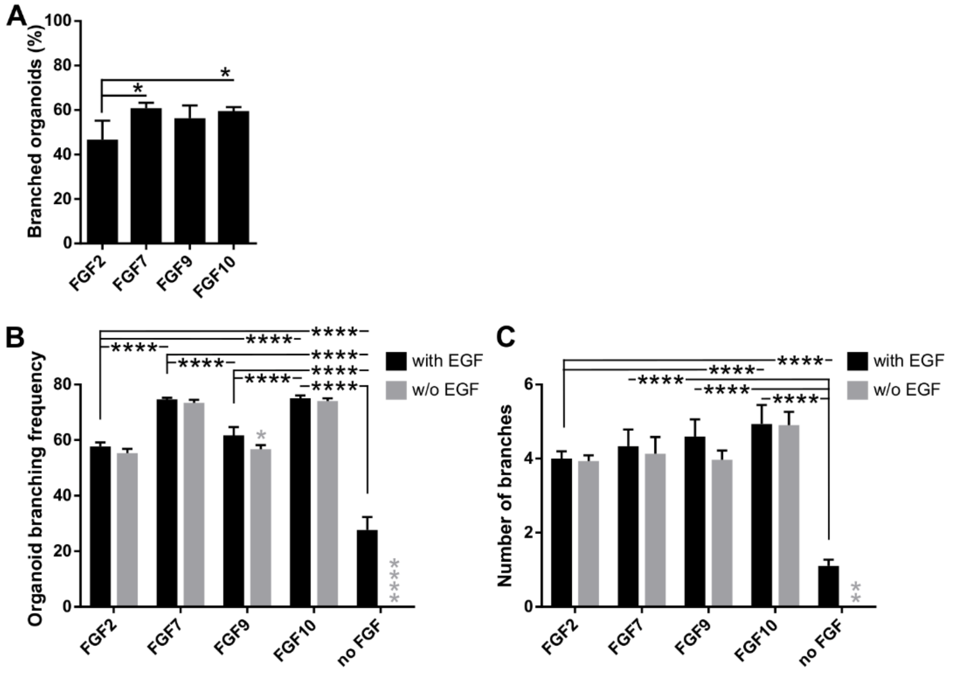

Supplementary Figure 4. FGFs promote branching of lung organoids. (**A**) Branching efficiency (%) of lung organoids on day 14 of culture, shown as mean + SD; n = 3-4. *P<0.05 (one-way ANOVA). (**B, C**) The plots show organoid branching frequency (**B**) and number of branches (**C**) of organoids formed in media with different FGFs and with or without EGF as indicated. The plots show mean + SD; n = 3, N = 15-20 organoids/treatment. The grey symbols indicate significance between the culture with and without EGF for the respective FGF. *P<0.05; **P<0.01; ****P<0.0001 (two-way ANOVA).

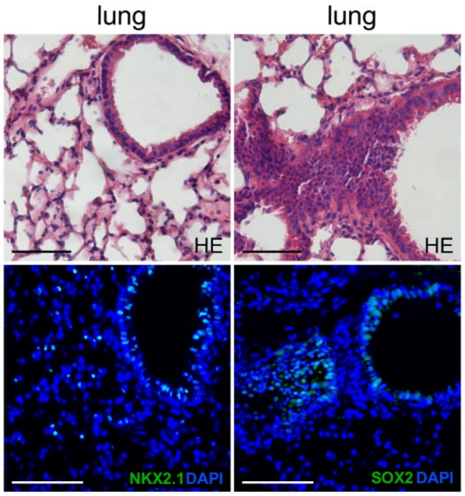

Supplementary Figure 5. Lung tissue staining as a positive control for immunofluorescence analysis of SOX2 and NKX2-1 in lung organoids. Paraffin sections of lung from 6 weeks old mouse were stained with hematoxylin and eosin (H&E; top panel) or by immunofluorescence for markers NKX2-1and SOX2 (bottom panel). Blue, nuclei (DAPI). Scale bars, 100 µm.

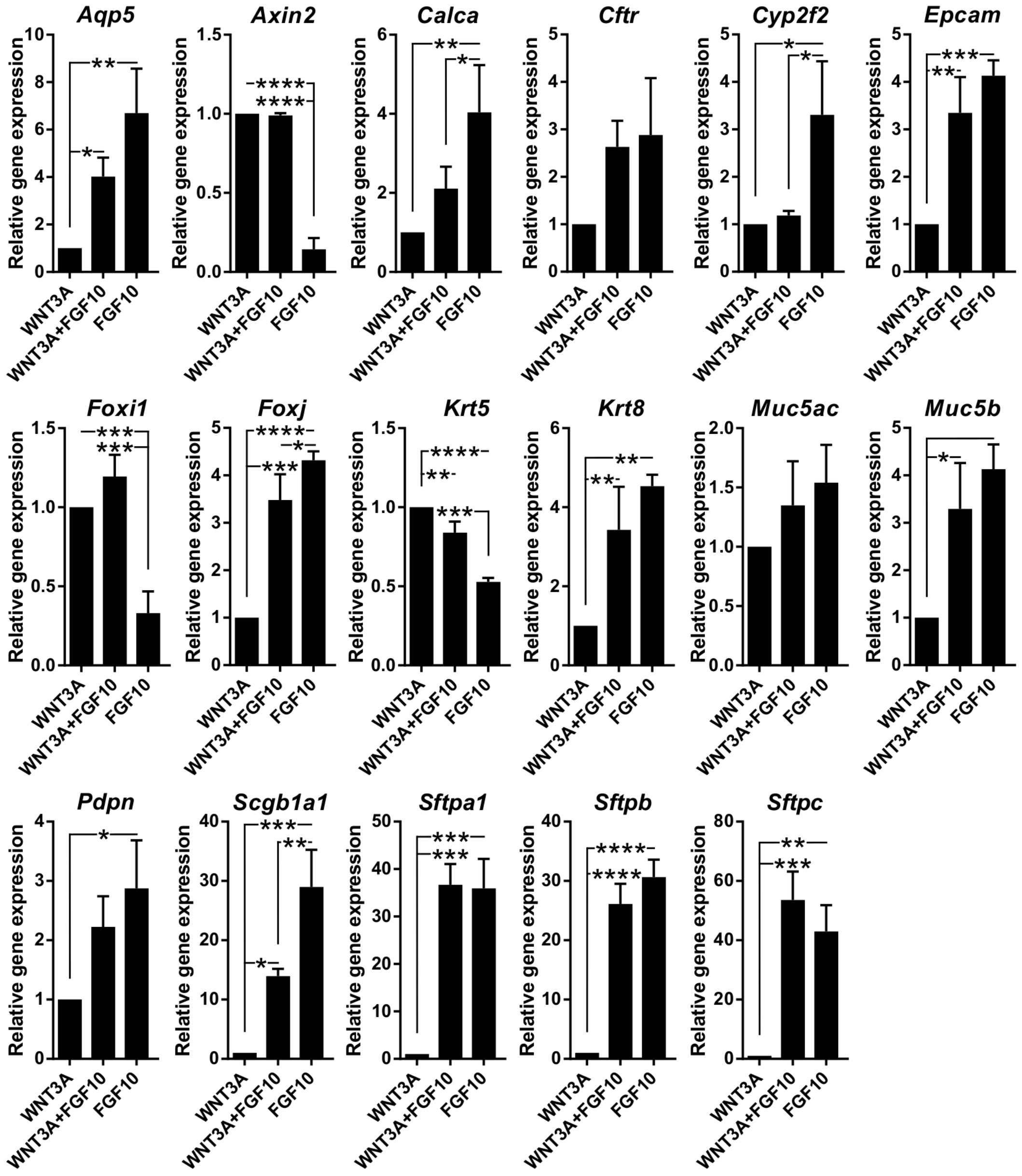

Supplementary Figure 6. FGF10 promotes organoid differentiation. (**A**) Results from qPCR analysis of expression of candidate genes in organoids grown with WNT3A, WNT3A and FGF10, or FGF10. The plots show mean + SD, n = 3. The stars indicate statistical significance between WNT3 and WNT3A+FGF10. *P<0.05; **P<0.01; ***P<0.001; ****P<0.0001 (one-way ANOVA). The plot is extended version of the plot in Figure 6H.

Supplementary Tables

Supplementary Table 1. Overview of antibodies used in this study.

Antigen	Clone	Catalog #	Conjugate	Host	Supplier	Dilution
**Flow cytometry:**						
CD24	M1/69 monoclonal	48-0242-82	eFluor 450	Rat	eBioscience	1:1000
CD45	30-F11 monoclonal	25-0451-82	PECy7	Rat	eBioscience	1:1000
CD49f	GoH3 monoclonal	12-0495-82	PE	Rat	eBioscience	1:1000
CD104	346-11A monoclonal	123608	Alexa Fluor 647	Rat	BioLegend	1:1000
EpCAM	G8.8 monoclonal	11-5791-82	FITC	Rat	eBioscience	1:1000
7-AAD	N/A	559925	IP	N/A	BD Biosciences	1:100
**Immunofluorescence:**						
Keratin 5	Poly19055 polyclonal	905504	Unconjugated	Rabbit	BioLegend	1:200
Cytokeratin 8	1E8	904804	Unconjugated	Mouse	BioLegend	1:200
Keratin 14	Poly19053 polyclonal	905304	Unconjugated	Rabbit	BioLegend	1:200
E-cadherin	M168 monoclonal	ab76055	Unconjugated	Mouse	Abcam	1:200
Prosurfactant protein B	Polyclonal	ab15011	Unconjugated	Rabbit	Abcam	1:200
Prosurfactant protein C	Polyclonal	AB3786	Unconjugated	Rabbit	Millipore	1:200
CC10	T-18 polyclonal	sc-9772	Unconjugated	Goat	Santa Cruz	1:200
Aquaporin 5	G-19 polyclonal	sc-9890	Unconjugated	Goat	Santa Cruz	1:100
MUC5AC	45M1 monoclonal	MA5-12178	Unconjugated	Mouse	Thermo Fisher Scientific	1:100
Aceylated α tubulin	6-11B-1 monoclonal	sc-23950	Unconjugated	Mouse	Santa Cruz	1:100
Lysozyme	EPR2994(2) monoclonal	ab108508	Unconjugated	Rabbit	Abcam	1:200
TTF1 (NKX2-1)	EP1584Y monoclonal	ab76013	Unconjugated	Rabbit	Abcam	1:400
SOX2	Polyclonal	ab97959	Unconjugated	Rabbit	Abcam	1:400
β-catenin	12F7 monoclonal	sc-59737	Unconjugated	Mouse	Santa Cruz	1:200
Rabbit	N/A	A-11008	Alexa Fluor 488	Goat	Life Technologies	1:800

Rabbit	N/A	A-11036	Alexa Fluor 568	Goat	Life Technologies	1:800
Goat	N/A	A-11055	Alexa Fluor 488	Donkey	Life Technologies	1:800
**Antigen**	**Clone**	**Catalog #**	**Conjugate**	**Host**	**Supplier**	**Dilution**
Goat	N/A	A-11058	Alexa Fluor 594	Donkey	Life Technologies	1:800
Mouse	N/A	A-11031	Alexa Fluor 568	Goat	Life Technologies	1:800

Supplementary Table 2. The primers used for qPCR.

Gene	Forward primer	Reverse primer	Length [bp]
Actb	GGCTGTATTCCCCTCCATCG	CCAGTTGGTAACAATGCCATGT	154
Aqp5	AGAAGGAGGTGTGTTCAGTTGC	GCCAGAGTAATGGCCGGAT	220
Axin2	TGACTCTCCTTCCAGATCCCA	TGCCCACACTAGGCTGACA	105
Calca	GAGGGCTCTAGCTTGGACAG	AAGGTGTGAAACTTGTTGAGGT	101
Cftr	CCCTTCGGCGATGCTTTTTC	AAGCCTATGCCAAGGTAAATGG	166
Cyp2f2	GGACCCAAACCTCTCCCAATC	CCGTGAACACCGACCCATAC	106
Eef1g	TTCCTGCCGGCAAGGTTCCA	TGCCGCCTCTGGCGTACTTC	119
Epcam	GCGGCTCAGAGAGACTGTG	CCAAGCATTTAGACGCCAGTTT	139
Foxi1	CCTCTCCACCATGACAGCAT	TCCCATGGCTACTGAGGTTG	155
Foxj1	CCCTGACGACGTGGACTATG	GCCGACAGAGTGATCTTGGT	114
Krt5	CTCTGTCGTTACAAACAGTG	CTTAGCCCGCTACCCAAACC	159
Krt8	CAAGGTGGAACTAGAGTCCCG	CTCGTACTGGGCACGAACTTC	187
Muc5ac	GTGGTTTGACACTGACTTCCC	CTCCTCTCGGTGACAGAGTCT	103
Muc5b	TCCCTAGCATGAGCGCCTTA	CCACGACGCAGTTGGATGTT	178
Pdpn	ACCGTGCCAGTGTTGTTCTG	AGCACCTGTGGTTGTTATTTTGT	159
Nkx2-1	CGCCTTACCAGGACACCAT	GCCCATGAAGCGGGAGA	98
Scgb1a1	ATGAAGATCGCCATCACAATCAC	GGATGCCACATAACCAGACTCT	135
Sftpa1	GAGGAGCTTCAGACTGCACTC	AGACTTTATCCCCCACTGACAG	103
Sftpb	CTGCTTCCTACCCTCTGCTG	CTTGGCACAGGTCATTAGCTC	175
Sftpc	ATGGACATGAGTAGCAAAGAGGT	CACGATGAGAAGGCGTTTGAG	117
Sox2	GCGGAGTGGAAACTTTTGTCC	GGGAAGCGTGTACTTATCCTTCT	156
Sox9	GAGCCGGATCTGAAGAAGGA	GCTTGACGTGTGGCTTGTTC	151
